# Ornamental Plants and Urban Gardening

**DOI:** 10.3390/plants12244096

**Published:** 2023-12-07

**Authors:** László Orlóci, Albert Fekete

**Affiliations:** Institute of Landscape Architecture, Urban Planning and Garden Art, Hungarian University of Agriculture and Life Sciences (MATE), 1118 Budapest, Hungary

**Keywords:** ornamental plants, urban gardening, green areas, landscape architecture

## Abstract

Urban green areas serve both the mental and physical health of the people living in the settlements; therefore, the ornamental plants used on green areas currently have a prominent role in reducing the effects of climate change and urbanization, as well as in providing ecosystem services. This is a dynamically changing, new field that requires close cooperation with several scientific fields, such as landscape architecture and plant physiology, genetics, plant breeding, and ecology. The monitoring and research of settlement communities as ecological systems greatly serves the perception of the effects of climate change and helps to mitigate them. The sustainability and economic operation of established urban green space systems can be made effective by applying innovative technologies. The Special Issue “Ornamental Plants and Urban Gardening” was launched in 2022 and published 13 articles on the topic until 31 July 2023. The published articles also have a very wide spectrum of topics, which also shows the diversity and the interdisciplinary nature of the scientific field. In the following, we present the main topics of the published articles and the results with which their authors contributed to the enrichment of the scientific field. We present a brief summary of the articles in shorter subsections.

## 1. This Plant Application, Question of Urban Plants

Due to the development of new urban habitats, where traditional varieties cannot be adapted, the demand for the production of ornamental plant varieties with improved properties has increased [1]. At the beginning of the 20th century, plant biologists established that the frequency and efficiency of genetic modifications in treated seeds could be increased by using chemical and radiation technology [2]. Subsequently, various mutagens, such as physical or chemical mutagens, were used to induce a wide range of genetic variability, leading to and contributing to current plant breeding [3]. Thus, radiation-induced mutation breeding is a remarkable method to produce superior mutant varieties, in contrast to conventional breeding, such as selection and crossbreeding, which involves time- and labor-intensive, limited genetic trait changes [4,5,6]. A research article in the Special Issue was based on this topic; the article dealt with the mutational breeding of *Rudbeckia hirta*, and the results were proven by histological and stress measurements. Kisvarga et al. [7] stated that in the future, the breeding of varieties intended for urban environments that tolerate the changed climate, and with it the urban biotic and abiotic stress effects, will become an increasingly important factor. The Hungarian cultivars currently under development form a very important genetic basis, but they no longer meet the challenges of today. Mutation breeding, on the other hand, can mark a new direction and represent a new way to create varieties that can be used in urban public areas of the 21st century from old varieties. The Hungarian varieties no longer meet the requirements due to the effects of today’s climate, and the old varieties are not fashionable either—which is an essential aspect in the breeding of ornamental plants. Gamma radiation had a detectable and favorable effect on the *Rudbeckia* genome. Higher doses showed more favorable phenotypic characteristics. The difference between the generations was also noticeable, and in many cases the positive effect of the treatments remained.

Honfi et al. [8] also researched the issues of urban plant application; the use of *Limonium gmellini* subsp. *hungaricum* was investigated as a potential ornamental plant on secondary saline soils. The importance of those authors’ research is also shown by climate change, one of the consequences of which may be the salinization of the soil. It is estimated that the globe loses at least 0.5–1% of its arable land to salinization every year [9], and half of the world’s arable land could disappear by 2050 if current trends continue [10]. Primary symptoms of salt toxicity in plants include ion damage, osmotic stress, and oxidative stress [11]. These pressures negatively affect plant growth and development. Salinity can also cause physiological drought, which occurs when plant roots respond to environmental stimuli such as water scarcity or salt ion abundance in the soil [12]. *Limonium* sp. or recretohalophytes are characteristic halophytes that have special structures on their epidermis, such as salt vesicles and salt glands, which allow them coping with salt-rich environments [13]. During the measurements, the authors used sand and clay as the growing medium and examined the morphological and plant physiological properties of the plants at different salt concentrations. It was found that *Limonium gmelinii* subsp. *hungaricum* in clay soil was able to survive and grow better than in sandy soil despite an increasing salt concentration.

Szabó et al. [14] also examined urban vegetation, but from a different perspective. However, the basic concept here was also the ecological changes that occur during climate change. A common question in landscape architecture and horticultural practice is the adaptability and applicability of plant species with different conservation status (protected, highly protected and invasive) [15]. Invasive species, which often start their careers in botanical gardens, have the opposite effect on nature conservation. Due to their high reproductive patterns and aggressive spread, plants released from gardens can infect large areas, causing severe ecological and economic damage. Control is often a problem, even in intensively managed plantations. Observations were made in Hungary, in the Buda Arboretum. The authors established that, based on the experience observed and collected in the garden, non-native species introduced into the urban environment as integrated elements can increase the aesthetic and recreational benefits [16], as well as have a positive ecological effect on urban green spaces [17]. Invasive plants can cause a decrease in biodiversity [16], a decrease in ecosystem resilience to disturbances, and ecosystem degradation [18]. The introduction of invasive or potentially invasive taxa by botanic gardens or cultivation in nurseries can also trigger deliberate invasion [19], so plant collections have a huge responsibility for future plant use proposals.

The article of Yessoufou [20] is also related to this field. The main topic of the work is the intraspecies changes in nectar sugar mass along phylogenetics, which distinguish native plants from non-native plants in urban green areas. The author carried out his research in the south of England. The topic is relevant since the function of urban green spaces as the service of nectar production, which is related to the diversity of pollinators found in urban areas, is important but not sufficiently debated. A recent study showed that although urban green spaces do not produce quantitatively more nectar than farmland and nature reserves, the sources of nectar supply in green spaces are more diverse, mostly caused by non-native plants. Indeed, compared to other systems, urban gardens provide 85% of the urban plant nectar supply [21], but it remains unclear whether nectar supply can be demonstrated as a “hidden or non-obvious” criterion behind human selection of non-native plants. The authors investigated whether functional traits integrated into a phylogenetic framework could reveal the subtle criteria underlying the introduction of non-native plants into urban green spaces. No differences were found in functional traits between natives and non-natives. There was also no evidence that functional traits predicted nectar production, regardless of how nectar production was measured. In their measurements, the authors found that the average sugar concentration of nectar per flower is evolutionarily distributed both within closely related non-native plants and within nearby native plants. However, phylogenetically close species show similar intraspecific variation in nectar sugar mass per flower, but this is only true for non-native plants, thus revealing non-obvious selection criteria for non-native plants in urban green spaces. The results show that the phylogenetic pattern of within-species variation in nectar sugar mass per flower is the main criterion for distinguishing non-native from native plants in urban green spaces in southern England. The study suggests that differences in nectar production patterns not only affect human selection criteria for non-native species that can be selected and introduced into urban green spaces, but, pending further investigation, may also be good candidates for ecological services that may predispose non-native species to colonize.

The study of Ye [22] about turmeric was also related to the topic, which is very important in terms of both climate change and urbanization. His research will facilitate cross-species hybridization and the introduction of genetic variation from wild species into turmeric varieties in the future, which may be useful for sustainable employment in urban green spaces. Interspecific or intergeneric hybridization is one of the main methods for breeding new varieties in plants [23]. There are no reports on the fertility of interspecies hybrids of Turmeric, even though it is an excellent ornamental and edible herb with promising market potential. Until now, a large number of excellent varieties have been obtained by interspecific hybridization [24,25]. Parental fertility is one of the main factors affecting the seeding rate of interspecific hybridization [26]. The incompatibility of hybridization can be easily overcome by using species or cultivars with strong stigma susceptibility as female parents. Selecting cultivars with high pollen vigor or germination rate as male parents can significantly increase fruit set rate [27]. Meanwhile, the ploidy level and chromosome number of the parents can also affect the compatibility of the parents [28]. Whether chromosome numbers are significantly related to fertility is still unknown, and the question needs to be further investigated by studying chromosome compression and karyotype analysis. Based on hybrid identification results, all individuals from the four combinations showed male-specific bands, indicating that the true hybrid ratio of the crosses was 100%. The results will facilitate interspecies hybridization and the introduction of genetic variation from wild species into turmeric cultivars in the future, which could be useful in realizing sustainable application in urban green spaces.

## 2. Landscape Architecture and Green Areas (8 Articles)

The topic was introduced by guest editor and co-author Albert Fekete with his article [29], the topic of which was the 17–18th century plant application in the late Renaissance Transylvania. The topic is extremely important, as it is a great challenge for landscape architecture to take historical values into account and integrate them with new functions and uses, as well as the latest demands for improving water management, energy transition, and creating a comfortable and healthy living environment for people. During their investigation, the authors identified 81 late Renaissance residence gardens in Transylvania based on archival and literary sources as well as field studies.

It was established that in the garden history of Transylvania, the late Renaissance style spread almost a hundred years later than in other parts of Europe. Nevertheless, in terms of the number of gardens in Transylvania, the Renaissance can be called the leading garden style in this part of the country. The number of late Renaissance gardens is much higher than the number of early Renaissance or later Baroque gardens. This is the consequence of Transylvania’s political and economic independence in the 17th century [30,31,32,33,34,35,36,37,38,39].

The history of the late Renaissance gardens in Transylvania is primarily about economic sustainability. For this reason, mixed gardens often appear (ornamental and vegetable gardens, and even orchards together), although in many places the various kitchen and vegetable gardens or orchards are also represented separately.

Given the condition of what remains of these historical artefacts, restoration in the strict sense is impossible. Destruction, missing archival sources, change in ownership and sustainability reasons further complicate the restoration work.

In addition to parks, urban cemeteries are also very important in urban ecosystem service and habitat protection. The topic was discussed in the Special Issue in the article of Sallay et. al. [40]. Similar to city public parks, cemeteries are an important part of the urban ecosystem; they provide a semi-natural habitat for many species of plants and animals, as well as a wide range of ecosystem services: they improve air quality, reduce the urban heat island phenomenon, and have aesthetic and recreational value. This article explores the role of cemeteries in the green infrastructure network beyond their sacred and commemorative roles and their importance as habitats for urban flora and fauna. It has been found that cemeteries can contribute to the preservation of biodiversity in cities. The role of dead trees in habitat conservation was highlighted. Dead, standing tree trunks are usually absent from cemeteries, even though they are particularly important habitats for hundreds of species. Habitat diversity should be increased, and traditional maintenance should be reduced by decreasing the mowed areas, the frequency of mowing and the use of herbicides, and the creation of more complex habitats, which may include new forests.

The issue of allergenic plants is a particularly important topic of urban plant application and landscape architecture planning, on which Magyar [41] wrote an article. The topic is extremely important, as plants are often used in allergy-related medicinal products and services, and pollen from many plant species can cause allergic disease (pollinosis) in sensitive people [42]. In the last two decades, the incidence of sensitization to pollen has increased from 13% to 30% [43]. Demonstration of allergenic plants is an important tool for patient education, contributing to the prevention of pollinosis, as patients can recognize the plants and avoid pollen exposure. The aim of this study was to evaluate the pictorial content of allergy-related web pages depicting plants. A total of 562 different photographs depicting plants were collected using an image search engine, identified and categorized according to their potential allergenicity. Out of a total of 124 plant taxa, 25% of the plants were at the genus level, and another 68% were at the species level. Plants with a low allergenic effect were included in 85.4% of the images, while plants with a high allergenic effect were only included in 4.5% of the image information. *Brassica napus* was the most frequently identified species (8.9% of all plants identified), while flowering *Prunoidae*, *Chrysanthemum* spp. and *Taraxacum officinale* was also common. Considering both the allergological and planning aspects, some plant species were recommended for more professional and responsible advertising. The Internet can provide visual support for patient education about allergenic plants, but emphasis must be placed on conveying the correct visual message. Appropriate illustrations help patients recognize and avoid allergenic plants and reduce exposure to allergenic pollen.

Also related to landscape architecture and the role of trees was the article of Nádasy et al. [44], the topic of which is spatial composition aspects in relation to the importance of trees in the urban landscape. Individual trees and tree compositions provide a wide range of cultural ecosystem services, including playing a key role in defining the character of the city. In Hungary, the tools for urban landscape protection have recently been expanded, thus putting the topic in the spotlight. However, the importance of natural elements (and especially trees) in relation to the urban landscape is still little researched. In this article, a new methodology is used to analyze and evaluate the character-shaping significance of the trees and wood compositional elements that define the urban character of the historical gardens in Hungary. In summary, the results show that the trees associated with historic gardens have a diverse and dynamically changing impact on the urban landscape, which has recently played an increasingly important role in the protection of local cultural heritage. The factors behind the visual impact on wooden elements and the different types of visibility that can help categorize and preserve the cultural services provided by these living pieces of cultural heritage have been identified. The methods and results are not country specific and can be considered broadly applicable and globally relevant. Research related to the inclusion of trees associated with historical gardens and parks in the Hungarian urban landscape protection toolkit suggests that recognition of trees as a visually significant element is lacking even within existing frameworks. While urban landscape protection regulations are implemented to varying degrees around the world, the professional recognition of objects as valuable is key to ensuring their survival and maintenance.

Teichmann et al. [45] published work on the role of schools in the Special Issue. Their more specific topic was cooperation between schools for the development and implementation of schools’ self-constructed greening systems. However, greening, which improves indoor climate and people’s well-being, is integrated to a very limited extent in public institutions such as schools. The reasons for this are seen in the lack of knowledge and financing opportunities. Among other things, the focus of the MehrGrüneSchulen research project is the interdisciplinary development of cost-effective greening solutions for schools. During the study, in the interdisciplinary cooperation of a technical school and a horticultural school, a total of twelve low-cost greening systems were developed for school indoor and outdoor spaces, six of which were implemented and greened in the construction yard of the technical school. In addition, the structural implementation of the adapted student plans took place in another nine schools across Austria, where the school classes were involved in the construction and on-site greening. In this way, they promote the construction of green infrastructure in Austrian schools, and at the same time promote climate-friendly construction, the development of social and moral skills, and connection and interaction with nature. This contributes to the gradual improvement of air quality and microclimatic conditions in and around schools and can increase the acceptance and awareness of building greening.

Dominici et al. also wrote about cost-effective greening methods in their article [46]. Due to the increasing lack of space in urban areas, vertical greening systems (VGS) are becoming increasingly popular as a means of increasing urban greening with building facades. VGSs are usually installed and managed by experts due to their technical complexity; however, the role of local communities is becoming increasingly important through the practice of do it yourself (DIY). The results of this study contribute to reducing the lack of do-it-yourself technical information related to the design choice and installation of VGS and indicate critical considerations that may arise when implementing VGS in small-scale urban spaces. UN SD Goal 11 encourages a growing trend and common interest in urban vertical greening, which must be supported by appropriate knowledge for beginners. Spreading knowledge about the importance and construction of VGSs as green infrastructure plays a key role in community engagement in more vertical greening and in promoting a participatory transition towards more sustainable and greener cities.

The article of Zhang et al. [47] was about coastal green roofs. Rapid urbanization and the growing demand for sustainable development have led to the emergence of green roof landscapes in oceanic cities. These roof gardens provide many environmental benefits and contribute to the overall well-being of city dwellers. However, optimizing the design and interaction experience of green roof landscapes requires the integration of intelligent technologies. The applied 3DMAX modeling and application of VR technology enriches the design process by providing immersive experiences for designers, users and stakeholders. By simulating and visualizing rooftop gardens, designers can intelligently interact with the space and make well-informed decisions to effectively improve landscape design. The compatibility between computer vision and green roof landscape design in maritime cities underscores the potential of these technologies to create aesthetic and functional green spaces.

The research topic of Yuan et al. [48] was similarly focused on coastal cities. The purpose of this paper was to study the design of maritime urban (MU) botanical landscapes based on computer vision technology (CVT) and multimodal interaction design (MID) theory, so that the design of MU botanical landscapes can meet the needs of people’s psychological and visual behavior while allowing participation and experience of the landscape to better meet people’s viewing, leisure and entertainment needs. Based on the theory of MID, this paper explores the application of human-to-human and human-to-landscape communication and interaction in MU installation design and attempts to explore and summarize content and methods, the interactive LD in maritime cities with theoretical foundations and research value. The aim was to raise the theoretical level of interactive LD on the one hand, and to serve as a new reference for future maritime city (MC) LD on the other. At the level of practical application, in the field of LD, the new concept of computer vision is introduced to fully understand people’s visual needs and increase the practicality and pleasantness of the MU landscape, hoping to attract more people to play and relax. Due to the attractiveness of MU landscapes to tourists, the planning and execution of the landscape no longer focuses on its appearance, but on the participation and experience of people. The study shows that as a designer, one must be able to analyze and solve the problems of the landscape from the audience’s perspective, and one must be able to satisfy people’s physical and mental needs for decorative, interactive and botanical landscape content enrichment.

The Special Issue covered very important and current topics, the diversity of which gives an idea of the fact that we need to continue researching this new field of science, since the ever-increasing urbanization and climate change demand this. I thank the editors and the authors of the articles for their joint work.
